# Clinical Practice Guidelines in an Era of Accountability, Saudi Arabia: A Call for Action

**DOI:** 10.1007/s44197-023-00135-y

**Published:** 2023-07-14

**Authors:** Hoda M. Abdellatif, Amani Al-Muallem, Afaf Saleh Almansoof, Sami A. AlRohaily, Abdullah Alzahrani, Hussah AlGhodaier, Mohammad Saeedi, Nahar AlAzemi, Imad Hassan

**Affiliations:** 1 Preventive Dental Sciences, College of Dentistry—Princess Nourah Bint Abdulrahman University, Riyadh, Saudi Arabia; 2 Family Medicine, King Abdulaziz Medical City, Riyadh, Saudi Arabia; 3 Rehabilitation, King Abdullah Specialized Children Hospital, Ministry of National Guard—Health Affairs, Riyadh, Saudi Arabia; 4 Family Medicine Academy, Ministry of Health, Madinah, Saudi Arabia; 5 National Center for Evidence-Based Medicine, Saudi Health Council, Riyadh, Saudi Arabia; 6 Saudi Health Council, Riyadh, Saudi Arabia; 7 Department of Medical Protocol, King Abdulaziz Medical City, Riyadh, Saudi Arabia; 8 Public Health Sciences, Texas A&M University College of Dentistry, Dallas, TX USA

**Keywords:** CPG appraisal, Evidence-based medicine, Saudi Health Council, Holistic Appraisal Tool for Clinical Practice Guidelines

## Abstract

**Introduction:**

Clinical Practice Guidelines (CPGs) development and implementation in the Kingdom of Saudi Arabia are suboptimal. The Kingdom’s Vision 2030 envisages a transformational change to achieve an effective, integrated, value-based ecosystem focused on patient health.

**Objectives:**

This study aimed to develop a CPG appraisal tool that will support the realization of the Kingdom’s Vision 2030 through the development of high-quality and highly implementable CPGs. To maximize its impact, all vital healthcare paradigms, such as systems thinking, value-based healthcare, and information technology, will robustly be incorporated in the tool.

**Methods:**

The Saudi Health Council through its National Center of Evidence-Based Medicine (NCEBM) embarked on a program to develop this appraisal tool. A taskforce of experts was selected based on their experience in evidence-based practice and training. The task force, through a methodology of extensive literature review, deliberation, outside experts’ feedback, and Delphi and consensus voting, developed a prototype appraisal tool that was named the Holistic Appraisal Tool for CPGs (HAT-CPG).

**Results:**

The HAT-CPG was developed comprising three sections: an initial basic information section, an internal validity section, and an external validity section with a total of 13 section items and 73 reporting elements.

**Conclusion:**

It is envisaged that the Holistic Appraisal Tool will support CPG developers and users in Saudi Arabia in realizing the objectives for which it was developed.

## Saudi Arabia Healthcare System Transformation

Vision 2030, Saudi Arabia’s national growth and economic development strategy, calls for the country’s reliance on oil to be reduced, its economy to be diversified, and public service sectors such as health and education, as well as infrastructure, recreation, and tourism, to be strengthened. The Kingdom of Saudi Arabia’s (KSA) healthcare system is set to undergo major changes under Vision 2030. The healthcare transformation initiative seeks to “restructure the Saudi health sector in order to improve its status and capacities as an effective, integrated, value-based ecosystem focused on patient health.” These objectives will also be met by implementing new healthcare paradigms and promoting public health and disease prevention [1]. To meet the needs of an ever-growing population, the healthcare system must be completely overhauled.

The Kingdom’s current critical issues, including rising healthcare spending [2], an aging population, and an increase in the incidence of non-communicable diseases [3], highlight the need for such a change. The high levels of dissatisfaction among healthcare users, as evidenced by patient complaints [4–6], increased harm to patients [7–9], healthcare providers’ burnout [10], and lawsuit [11] concerns, exacerbate these issues.

## Clinical Practice Guidelines

Evidence-based healthcare recommendations provided by well-developed clinical practice guidelines (CPGs) can benefit all stakeholders—from policymakers and healthcare workers to patients and healthy individuals. According to the Institute of Medicine, CPGs are statements that include recommendations based on a systematic review of evidence and an assessment of the benefits and drawbacks of alternative care options that are designed to optimize patient care [12]. They contribute to the development of clinical policy and the widespread use of evidence throughout the healthcare system and help improve clinical decision-making in the context of patient care [13]. Although CPGs play an important role in health policy development, they are designed to cover a wide range of healthcare topics (e.g., health promotion, screening, diagnosis, therapy, etc.) [13]. However, the quality of CPG recommendations varies greatly, and some fall short of the basic standards and may even be harmful or inaccurate [14–16].

Healthcare practitioners in the United Kingdom follow CPGs developed and/or approved by the National Institute for Health and Care Excellence (NICE) or the Scottish Intercollegiate Guidelines Network (SIGN), while practitioners in Germany follow the Association of Scientific Medical Societies (AMWF), and those in Canada follow the International Centre for Evidence-Based Medicine Canada. No such national reference exists for health practitioners to follow in Saudi Arabia. Depending on where they were trained, healthcare practitioners in the KSA use a variety of CPGs. This variation in healthcare management has resulted in inconsistencies in the quality of healthcare for the country’s most prevalent and costly diseases such as diabetes, hypertension, and ischemic heart disease. Furthermore, due to factors such as lifestyle, culture, and religious values, these guidelines may not always address the appropriate care required for the Saudi community and residents.

## Challenges and Solutions

CPGs’ quality and implementation in the Gulf States and Saudi Arabia have been deficient [17]. Despite significant advances in medical science and the availability of numerous CPGs, high-quality healthcare and the anticipated outcomes have yet to be realized [18, 19].

More than 40 CPG appraisal tools have been developed to date to accelerate CPG development, credibility (internal validity), and applicability (external validity) [20, 21]. Despite the availability of these tools, CPGs’ quality and implementation remain suboptimal [22, 23]. However, it is important to note that most guideline evaluation tools focus on the methods used to generate guidelines rather than on how they should be implemented.

Implementation issues are caused by a variety of factors. Two factors contribute to this weakness in CPGs: trustworthiness (credibility) and implementation (applicability) [24–27]. Guidelines will be of limited use if they are not put into meaningful practice. This is especially true if they were not created with the goal of effective implementation in mind [28–30]. While a CPG score derived from appraisal tools may appear high, its actual impact on practice may be minimal or non-existent [31]. Current CPGs clearly have a significant weakness in terms of implementation [32]. The evidence, on the other hand, clearly indicates that CPGs that include implementation tools have a higher success rate of raising standards of care [33–35]. CPGs should therefore include all relevant implementation tools in their guidance for solving or resolving healthcare challenges. Four components of the healthcare system should be addressed and targeted (organization/healthcare setting, healthcare professionals, patients, and the community). Each system element has a relevant and evidence-based implementation tool or tools. Multifaceted interventions outperform isolated interventions in terms of effectiveness [12]. Moreover, tools that target non-healthcare systems may be just as important, if not more so, than those that do. Accordingly, an assessment tool that considers CPG implementation at all levels of healthcare, as well as non-healthcare system interventions, is required. It should also uphold the moral and cultural values of the people it serves. Adherence to certain sociocultural aspects aids the successful implementation of CPGs [36].

## Call for Action

The Saudi Health Council (SHC) is a government organization authorized to coordinate all healthcare provider programs in Saudi Arabia. Its goal is to develop and implement healthcare service standards by managing and coordinating health communities’ programs. The SHC’s vision is to serve as a model for a world-class Saudi health system that improves the Saudi people’s health in a scalable, consistent, and responsible manner. The SHC has established the National Center for Evidence-Based Medicine (NCEBM), which is dedicated to developing evidence-based health practices. The center’s mission is to serve as the health system’s approved national reference center for knowledge and scientific evidence to support the development of more effective health practices.

One of the NCEBM’s pioneering goals was to develop a CPG appraisal tool that outperformed current appraisal tools while being tailored to the needs of the Saudi population. More importantly, the new CPG appraisal tool was intended to advise and support CPG developers and users in the KSA by providing them with practical leverage tools critical to achieving the 2030 healthcare transformation initiative.

Existing appraisal tools have been modified and reformed into the Holistic Appraisal Tool for Clinical Practice Guidelines (HAT-CPG), which examines the methodological rigor, transparency, and implementation actions required to develop a guideline.

## Holistic Appraisal Tool for Clinical Practice Guidelines Appraisal Tool

The HAT-CPG was created to increase the likelihood of CPGs being implemented successfully by drawing attention to issues that should be considered when appraising or developing guidelines. Two areas of focus were chosen. The first was a greater emphasis on implementation. The second was an innovative scoring system expected to improve the objectivity of this critical component. It is important to note that current CPG appraisal tool scoring systems and final scores, such as those obtained with the Appraisal of Guidelines for Research and Evaluation (AGREE) II tool, have no bearing on the success or failure of a guideline and its implementation as they focus on the methodological quality of CPGs. The HAT-CPG’s internal and external validity sections include nine paradigms that characterize modern healthcare: evidence-based practice, quality-based practice (patient safety), competency-based training and certification, value-based healthcare, patient- and family-centeredness, high-reliability organization principles, health information technology, artificial intelligence, and sociocultural acceptance. Furthermore, the CPG appraisal assessment includes critical and appropriate dissemination, implementation, and monitoring tools relevant to these paradigms, which are intended to strategically enlighten and educate CPG developers and users about these tools, thereby promoting their incorporation and successful implementation in modern CPGs. Table 1 illustrates some of the tools. The HAT-CPG is currently undergoing extensive validation. It is expected and planned that the tool will be complemented by a robust hands-on educational program on the tool’s various aspects and paradigms.

**Table 1:** Examples of elements incorporated into the Holistic Appraisal Tool for Clinical Practice Guidelines, Saudi Arabia

Dissemination *A comprehensive plan and list of dissemination tools for all stakeholders and users of the guideline at all levels (organizational, individual, population, *etc*.)*	• Tools for dissemination to healthcare providers, end users, stakeholders, patient support groups, public or community presentations (e.g., opinion leaders, academic detailing, and educational outreach), media releases, RSS feeds, phone text messaging (SMS), internet telephone, intelligent agents, etc • Development of patient and public editions highlighting the major recommendations of the guideline in the respective target population’s language
Implementation *Organization-level interventions*	• Necessary legislation, policies, and procedures • Need for inter-facility/integrated care units and centers of excellence • Clinical decision support and reminder systems (paper-based and electronic): clinical pathways, protocols, order sets, checklists, etc • System redesign (e.g., development of local knowledge translation committees, electronic health records, clinical practice units, care teams, advanced health practitioners, monitors, etc.)
Implementation *Incorporation of modern paradigm concepts and tools in the implementation advice*	*Explicit mention of relevance of the implementation tools to the concepts of:* • Value-based healthcare • Patient/family-centeredness • Quality/safety/reliability • Sociocultural and political system acceptance
Implementation *Non-healthcare system interventions and preventative strategies*	*Inclusion of actions by relevant sectors* • Actions related to population, community, NGOs, self-help groups, etc • Non-health body interventions: education, media, transport, housing/civil engineering, environment, agriculture, food industry, sport, entertainment, etc
Implementation *Mitigating actions*	• Interventions for mitigating any unintended consequences from the guideline implementation
Monitoring *Outcome and process criteria for monitoring implementation*	• Process criteria: Criteria to assess guideline implementation and adherence to recommendations • Outcome(s) criteria: Criteria for assessing the impact of implementing the recommendations • Timing: Advice on the frequency and interval of measurement • Mechanisms/tools: Operational definitions of how the criteria should be measured

## Conclusion

The NCEBM is dedicated to developing evidence-based CPGs. To accomplish this, the newly developed HAT-CPG appraisal tool is expected to help advise and support CPG developers and users in the KSA. The HAT-CPG was created to draw attention to issues that should be considered when evaluating or developing CPGs. It aims to increase the likelihood of CPGs being implemented successfully. This will help standardize healthcare delivery and management across the KSA based on the Saudi population’s sociocultural needs. The launch of the NCEBM and the development of the HAT-CPG can help achieve three strategic goals: 1) establish a national standard for health practice by approving and implementing evidence-based CPGs; 2) improve the quality of health services and patient safety by strengthening evidence-based health practices; and 3) support digital transformation in the field of evidence-based medicine.

## Data Availability

Not applicable.

## Abbreviations

AGREE: Appraisal of Guidelines for Research and Evaluation
AMWF: Association of Scientific Medical Societies
CPG: Clinical Practice Guidelines
HAT-CPG: Holistic Appraisal Tool for Clinical Practice Guidelines
KSA: Kingdom of Saudi Arabia
NCEBM: National Center for Evidence-Based Medicine
NICE: National Institute for Health and Care Excellence
SHC: Saudi Health Council
SIGN: Scottish Intercollegiate Guidelines Network

## References

[1] https://www.vision2030.gov.sa/v2030/vrps/hstp/. Accessed 3 Jan 2022.

[2] Alkhamis A, Hassan A, Cosgrove P (2014). Financing healthcare in Gulf Cooperation Council countries: a focus on Saudi Arabia. Int J Health Plann Manage..

[3] Herzallah HK, Antonisamy BR, Shafee MH, Al-Otaibi ST (2019). Temporal trends in the incidence and demographics of cancers, communicable diseases, and non-communicable diseases in Saudi Arabia over the last decade. Saudi Med J.

[4] Al Fraihi KJ, Latif SA (2016). Evaluation of outpatient service quality in Eastern Saudi Arabia Patient's expectations and perceptions. Saudi Med J..

[5] Al-Momani MM (2016). Gap analysis between perceptions and expectations of medical-surgical patients in a public hospital in Saudi Arabia. Med Princ Pract.

[6] Senitan M, Alhaiti AH, Gillespie J (2018). Patient satisfaction and experience of primary care in Saudi Arabia: a systematic review. Int J Qual Health Care.

[7] Aidah S, Gillani SW, Alderazi A, Abdulazeez F (2021). Medication error trends in Middle Eastern countries: a systematic review on healthcare services. J Educ Health Promot..

[8] Al Wahabi S, Farahat F, Bahloul AY (2017). Prevalence and preventability of sentinel events in Saudi Arabia: analysis of reports from 2012 to 2015. East Mediterr Health J.

[9] Aljadhey H, Mahmoud MA, Ahmed Y, Sultana R, Zouein S, Alshanawani S, Mayet A, Alshaikh MK, Kalagi N, Al Tawil E, El Kinge AR, Arwadi A, Alyahya M, Murray MD, Bates D (2016). Incidence of adverse drug events in public and private hospitals in Riyadh, Saudi Arabia: the (ADESA) prospective cohort study. BMJ Open..

[10] Rotenstein LS, Torre M, Ramos MA, Rosales RC, Guille C, Sen S, Mata DA (2018). Prevalence of burnout among physicians: a systematic review. JAMA.

[11] Almannie R, Almuhaideb M, Alyami F, Alkhayyal A, Binsaleh S (2021). The status of medical malpractice litigations in Saudi Arabia: Analysis of the annual report. Saudi J Anaesth..

[12] Institute of Medicine (US) Committee on Standards for Developing Trustworthy Clinical Practice Guidelines, Graham R, Mancher, M, Miller Wolman D, Greenfield S, Steinberg E (eds) (2011) Clinical practice guidelines we can trust. National Academies Press (US). 10.17226/13058 24983061

[13] Brouwers MC, Florez ID, McNair SA, Vella ET, Yao X (2019). Clinical practice guidelines: tools to support high quality patient care. Semin Nucl Med.

[14] Shaneyfelt TM, Mayo-Smith MF, Rothwangl J (1999). Are guidelines following guidelines? The methodological quality of clinical practice guidelines in the peer-reviewed medical literature. JAMA.

[15] Grilli R, Magrini N, Penna A, Mura G, Liberati A (2000). Practice guidelines developed by specialty societies: the need for critical appraisal. Lancet.

[16] Burgers JS, Fervers B, Haugh M, Brouwers M, Browman G, Phillip T, Cluzeau FA (2004). International assessment of the quality of clinical practice guidelines in oncology using the Appraisal of Guidelines and Research and Evaluation Instrument. J Clin Oncol.

[17] Koornneef E, et al. The development, implementation and evaluation of clinical practice guidelines in Gulf Cooperation Council (GCC) countries: a systematic review of literature. J Eval Clin Pract. 2015;21(6):1006–1013. 10.1111/jep.12337. 10.1111/jep.12337 25756849

[18] Cabana MD, Rand CS, Powe NR, Wu AW, Wilson MH, Abboud PA, Rubin HR (1999). Why don't physicians follow clinical practice guidelines? A framework for improvement. JAMA.

[19] Barth JH, Misra S, Aakre KM, Langlois MR, Watine J, Twomey PJ, Oosterhuis WP (2016). Why are clinical practice guidelines not followed?. Clin Chem Lab Med.

[20] Siering U, Eikermann M, Hausner E, Hoffmann-Eßer W, Neugebauer EA. Appraisal tools for clinical practice guidelines: a systematic review. PLoS One. 2013;8(12):e82915. 10.1371/journal.pone.0082915 PMC3857289 24349397

[21] Vlayen J, Aertgeerts B, Hannes K, Sermeus W, Ramaekers DA. Systematic review of appraisal tools for clinical practice guidelines: multiple similarities and one common deficit. Int J Qual Health Care. 200;17(3):235–242. 10.1093/intqhc/mzi027. 10.1093/intqhc/mzi027 15743883

[22] Hébert C, Watkins-Martin K, Ciquier G, Azzi M, Drapeau M (2021). The quality of six clinical practice guidelines in health and social sciences: are we on the right track?. Adm Policy Ment Health.

[23] Eikermann M, Holzmann N, Siering U, Rüther A (2014). Tools for assessing the content of guidelines are needed to enable their effective use–a systematic comparison. BMC Res Notes.

[24] Correa VC, Lugo-Agudelo LH, Aguirre-Acevedo DC, Contreras JAP, Borrero AMP, Patiño-Lugo DF, Valencia DAC (2020). Individual, health system, and contextual barriers and facilitators for the implementation of clinical practice guidelines: a systematic metareview. Health Res Policy Syst.

[25] Almazrou SH, Alfaifi SI, Alfaifi SH, Hakami LE, Al-Aqeel SA (2020). Barriers to and Facilitators of Adherence to Clinical Practice Guidelines in the Middle East and North Africa region: a systematic review. Healthcare (Basel).

[26] Keiffer MR (2015). Utilization of clinical practice guidelines: barriers and facilitators. Nurs Clin North Am.

[27] Correa VC (2015). Individual, health system, and contextual barriers and facilitators for the implementation of clinical practice guidelines: a systematic metareview. Health Res Policy Syst..

[28] Grimshaw J, Russell I (1993). Effect of clinical guidelines on medical practice: a systematic review of rigorous evaluations. Lancet.

[29] Grol R (2001). Success and failures in the implementation of evidence-based guidelines for clinical practice. Med Care.

[30] Davis DA, Taylor-Vaisey A (1997). Translating guidelines into practice: a systematic review of theoretic concepts, practice experience and research evidence in the adoption of clinical practice guidelines. CMAJ.

[31] Brouwers MC, Kho ME, Browman GP, Burgers JS, Cluzeau F, Feder G, Fervers B, Graham ID, Grimshaw J, Hanna SE, Littlejohns P, Makarski J, Zitzelsberger L (2010). AGREE II: advancing guideline development, reporting and evaluation in health care AGREE Next Steps Consortium. CMAJ.

[32] de Vasconcelos LP, Melo DO, Stein AT, de Carvalho HB (2021). Even high-quality CPGs seldom include implementation strategies. Front Pharmacol..

[33] Gagliardi AR, Brouwers MC, Bhattacharyya OK (2015). The development of guideline implementation tools: a qualitative study. CMAJ Open.

[34] Sabharwal S, Patel NK, Gauther S (2014). High methodological quality but poor applicability: assessment of the AAOS guidelines using the AGREE II instrument [published erratum in Clin Orthop Relat Res 2014;472:2309]. Clin Orthop Relat Res.

[35] Dobbins M, Hanna SE, Ciliska D, Manske S, Cameron R, Mercer SL, O'Mara L, DeCorby K, Robeson P (2009). A randomized controlled trial evaluating the impact of knowledge translation and exchange strategies. Implement Sci.

[36] Saha S, Beach C, Cooper L (2008). Patient Centeredness, Cultural Competence, and Healthcare Quality. JNMA.

